# How Affectively-Based and Cognitively-Based Attitudes Drive Intergroup Behaviours: The Moderating Role of Affective-Cognitive Consistency 

**DOI:** 10.1371/journal.pone.0082150

**Published:** 2013-11-14

**Authors:** Jie Zhou, John Dovidio, Erping Wang

**Affiliations:** 1 Institute of Psychology, Chinese Academy of Sciences, Beijing, China; 2 Department of Psychology, Yale University, New Haven, Connecticut, United States of America; University of California, San Francisco, United States of America

## Abstract

The moderating role of affective-cognitive consistency in the effects of affectively-based and cognitively-based attitudes on consummatory and instrumental behaviors was explored using two experimental studies in the intergroup context. Study 1 revealed that affectively-based attitudes were better predictors than cognitively-based attitudes regardless of affective-cognitive consistency for consummatory behaviors (e.g., undergraduates’ supportive behaviors toward government officials). Study 2, which investigated task groups’ supportive behaviors toward an immediate supervisory group, found that for these instrumental behaviors cognitively-based attitudes were better predictors than affectively-based attitudes only when affective-cognitive consistency was high. The present research also examined the mechanism by which affective-cognitive consistency moderates the relative roles of affectively-based and cognitively-based attitudes in attitude-behavior consistency. Results indicated that attitude-behavior consistency is eroded primarily because of the weaker relationship of affective or cognitive components to behaviors than to general attitudes. The reciprocal implications of research on attitudes and work on intergroup relations are considered.

## Introduction

Over the past seventy-five years, the study of intergroup relations has been one of the most active areas of social psychological research [1]. A substantial portion of that research has emphasized intergroup attitudes and ways to change them [2]. However, these attitudes only modestly predict discriminatory behaviors (meta-analytic *r* = 0.32 [3]; see also 4). Research on attitudes more generally has further emphasized the importance of considering the multi-componential nature of the attitudes – including affective, cognitive, and behavioral predisposition aspects – for understanding how attitudes guide behaviors [5,6]. The present work, drawing on research on attitude-behavior consistency more generally [7,8], investigated how, in the particular context of intergroup relations, the relationship between intergroup attitudes and behaviors may be moderated by the structural aspects of the attitudes and the nature of the behaviors being predicted.

Social psychologists have long been interested in identifying both properties of the attitudes and the nature of the behaviors that moderate the attitude-behavior relation [9]. Two relevant qualities of attitudes involve (a) the affective or cognitive basis of the attitudes [10-12], and (b) consistency between affective and cognitive components of the attitudes [13-15]. Attitude base, which refers to the relative weight of affect versus cognition in general attitudes [16,17], has been shown in previous research to be an important moderator of attitude-behavior consistency outside the domain of intergroup relations [18,19]. However, whether more affectively-based or cognitively-based attitudes are better predictors of behaviors depends on the nature of the behaviors in question.

Previous work has distinguished between two different types of behaviors: instrumental and consummatory behaviors. As originally defined by Millar and Tesser [20], instrumental behaviors performed to achieve a specific objective are steps toward a goal. Consummatory behaviors, by contrast, are appetitive or aversive behaviors performed for persons’ own sake. For instance, painting is instrumental if the painter does so only because he or she wants to earn money selling his or her works, and it is consummatory if the painter does so only because he or she is fond of painting per se [21]. In the context of social behavior, interpersonal preference is an example of consummatory behaviors used in previous research [22].

In general, attitude base (affective or cognitive) and the nature of the behaviors (instrumental or consummatory) are jointly critical in determining attitude-behavior consistency. Outside the domain of intergroup relations, two studies [7,20] manipulating attitude base and the type of behaviors found that affectively-based attitudes were associated more with consummatory behaviors, whereas cognitively-based attitudes predicted instrumental behaviors better. In the intergroup context, Dovidio, Esses, Beach, and Gaertner ([23]; see also 24) demonstrated that affectively-based attitudes toward outgroup were better predictors of willingness to make contact with outgroup members (a consummatory behavior), whereas cognitively-based attitudes were better predictors of endorsement of social policies for outgroup (an instrumental behavior).

In addition, consistency between affective and cognitive components of the attitudes (affective-cognitive consistency [25]), which represents the corresponding valence associated with cognitions about an attitude object and reported affective reactions [26-29], is another extensively researched moderator of the attitude-behavior relation. Attitudes reflecting greater affective-cognitive consistency better predict behaviors outside of the intergroup domain (e.g., between the attitudes toward volunteering for the psychological research and the behaviors as being the volunteer participants later [28]; between job satisfaction and work performance [29]).

Moreover, affective-cognitive consistency of the attitudes appears to further moderate the process in which attitude base and the nature of the behaviors influence the attitude-behavior relation. Millar and Tesser [30] directly explored the moderating role of affective-cognitive consistency in this process and found that consummatory behaviors (e.g., play the puzzle games for sake) were better predicted by affectively-based attitudes when affective-cognitive consistency was low, whereas instrumental behaviors (e.g., play the puzzle games for getting a high score in the following puzzle-solving test) driven by cognitively-based attitudes also occurred when affective-cognitive consistency was low. They further explained that when affective-cognitive consistency was high, affectively-based and cognitively-based attitudes were similar so that these attitudes related to all behaviors in a similar manner; while affective-cognitive consistency was low, affectively-based and cognitively-based attitudes were distinct so that these attitudes should relate differently to subsequent behaviors [30].

However, the evidence for this conclusion is mixed. Zhou, Wang, Dovidio, and Yu [31] in a study about consumers’ satisfaction and shopping behaviors (instrumental behaviors) manipulated attitude base and affective-cognitive consistency, and found that cognitively-based attitudes were better predictors of instrumental behaviors than affectively-based attitudes when affective-cognitive consistency was high, but cognitively-based and affectively-based attitudes exerted a roughly equal influence when affective-cognitive consistency was low. This study, however, did not examine consummatory behaviors.

The present research was designed to further investigate how the structural qualities of the attitudes and the nature of the behaviors combine to influence attitude-behavior consistency specifically in the intergroup contexts. Intergroup contexts may differ fundamentally from general attitude-behavior relationships because of the strong role that affect plays in intergroup relations. Thinking in terms of group identities spontaneously arouses emotions such as anxiety [32] and fear [33]. In addition, the affective components of intergroup attitudes are generally stronger predictors of intergroup behaviors than cognitive components [34]. Two different meta-analyses, one by Pettigrew and Tropp [35] examining the intergroup contact literature and another by Talaska et al. [4] reviewing attitude-discrimination research, both found that differential emotional reactions to ingroup and outgroup members (beyond overall positive and negative reactions) were significantly better predictors than cognitive measures (e.g., knowledge) of bias and discrimination.

Therefore, the current research, consisting of two studies, explored how attitude base, affective-cognitive consistency, and instrumental versus consummatory behaviors combine to influence attitude-behavior consistency specifically in the context of intergroup relations. Study 1 examined consummatory behaviors: it investigated undergraduates’ attitudes and supportive behaviors toward government officials. Study 2 focused on instrumental behaviors: it explored task groups’ attitudes and supportive behaviors toward an immediate supervisory group. Thus, this research examined the moderating role of affective-cognitive consistency in the effect of attitude base on the attitude-behavior relation respectively for different behaviors (i.e., consummatory and instrumental behaviors).

The present research potentially extends previous work in two main ways. First, the present research applies work on attitudes generally to the specific case of intergroup attitudes, for which affect plays a particularly strong role [34,35]. Intergroup attitudes differ from those nonsocial attitudes in a variety of ways [36]. People attend more strongly to social than nonsocial information [37], process social information more deeply [38], and show distinct patterns of learning and decision making with social and nonsocial cues [39]. Therefore, we explored the effects of affectively-based and cognitively-based attitudes and the potential moderating role of affective-cognitive consistency on the attitude-behavior relation in the intergroup domain.

Second, further emphasizing social processes, we assessed intergroup attitudes and behaviors from groups rather than from individual participants. Group attitudes were assessed by averaging the group members’ reports of the attitudes of the group as a whole, in an approach similar to that used for the measurement of team characteristics in the work-team context [40,41]. Group behaviors represented the group’s consensus after discussions that occurred within a limited timeframe [42-44]. Research on the interindividual-intergroup discontinuity effect [33] suggests that group-based responses have a particularly strong affective component and are less driven by “rationale” responses. In addition, because group attitudes (e.g., group emotional responses) are shared socially within a group they are likely to predict intergroup behaviors better than individuals’ attitudes [45].

Specifically, the present research investigated the potential moderating role of affective-cognitive consistency, which has been implicated for nonsocial attitudes [30], in intergroup attitudes. In Study 1 we tested in an intergroup context whether consummatory behaviors are generally better predicted by affectively-based attitudes [4,23,24,35] or only when affective-cognitive consistency is low [30]. In Study 2, we examined whether instrumental behaviors driven by cognitively-based attitudes occur in the condition of low affective-cognitive consistency [30] or in the condition of high affective-cognitive consistency [31].

## Study 1

The primary goal of Study 1 was to explore the moderating role of affective-cognitive consistency in the effects of affectively-based and cognitively-based attitudes on consummatory behaviors. We targeted undergraduates’ expressions of support toward government officials as consummatory behaviors because these expressions of preferences performed for sake did not materially advance the participant groups’ own goals and interests.

In this study, participant groups discussed what they felt and thought about government officials, along with other filler groups (i.e., teachers and doctors). They then responded to items representing their affective, cognitive, and overall evaluations to these groups. Measures of attitude base and affective-cognitive consistency were computed and related to expressions of support toward these groups.

Although, to our knowledge attitude base and affective-cognitive consistency have not been studied together in the intergroup context, previous research on intergroup relations revealed that the affective prejudice was more closely related to intergroup orientations than cognitive components [4,23,24,35,46]. Thus, we hypothesized that affectively-based attitudes toward outgroup would relate more strongly to intergroup consummatory behaviors than cognitively-based attitudes, possibly regardless of affective-cognitive consistency.

Moreover, the present study also explored the reasons why affective-cognitive consistency might not influence the effect of attitude base on attitude-behavior consistency for consummatory behaviors. In previous research, Lavine, Thomsen, Zanna, and Borgida suggested that affective and cognitive influences can differentially affect both attitudes and behaviors [47]. Therefore, to more fully understand how affect and cognition shape attitude-behavior consistency, we also evaluated their relationships to attitudes and behaviors separately.

### Method

#### Ethics Statement

All participants gave a written informed consent before this experiment, and their responses in the current study are all anonymous. In addition, this study was approved by the Institutional Review Board (IRB) of Institute of Psychology, Chinese Academy of Sciences.

#### Participants

Participants were 160 undergraduate volunteers (67 men and 93 women) at a Chinese university ranging in age from 18 to 27 years (mean = 21.65 years). They each received 20 yuan in exchange for their participation. Participants were randomly assigned to one of 40 groups, each of which consisted of four members [48]. 

#### Procedure

Upon arrival, participant groups were given a short oral introduction by a female experimenter. The introduction described the experiment as an investigation of “social perception and interaction.” Participant groups were then asked to discuss the presented topics within a limited timeframe and write down the group’s consensus without the use of a ballot (see 49).

Participant groups were given 20 minutes for discussion and were asked to make a list of the feelings that they experienced when they saw, met or thought about government officials, teachers, and doctors. They were also asked to record the typical characteristics exhibited or practiced by the members of these outgroups. The three outgroups were presented in a counterbalanced order using the Latin square design. Following the group discussions, participant groups’ attitudes toward three outgroups and the affective and cognitive components of these attitudes were measured. Participant groups’ supportive behaviors toward government officials, teachers, and doctors were obtained during another group discussion.

At the end of the procedure, each participant completed a paper-and-pencil questionnaire that examined his or her judgments about the experimental purpose of this study. None of the participants correctly identified the true purpose of the study.

#### Measures

To prevent participants from determining the focus of the present research, teachers and doctors were used as the filler groups. These two filler groups were chosen because teachers, doctors, and government officials typically receive favorable treatment and have comparatively high social status in China. 

The *affective components of group attitudes* toward the target group and two filler groups were assessed using three items based on an affective scale taken from Crites et al.’s study [10]. Participants responded to the question, “How does your group feel about government officials/teachers/doctors?” with the following three bipolar adjective pairs (alpha = 0.87): happy-unhappy, relaxed-angry, and loving-hateful. Answers were given on a four-point scale without a middle point. Group attitude was calculated by averaging the four members’ reported attitudes [40,41]; for government officials, the intraclass correlation coefficient (*ICC(2*)) indicating the variance among group members was 0.69 (*p* < .01).

The *cognitive components of group attitudes* were assessed using three items based on Group Perceptions Survey (see 12). Participants were asked to respond to the question, “What does your group think of government officials/teachers/doctors?” with the following three bipolar adjective pairs (alpha = 0.91): responsible-irresponsible, hardworking-lazy, and helpful-useless. Answers were given on a four-point scale without a middle point, and four group members’ reported attitudes were averaged to generate the index of group attitude. For government officials, the intraclass correlation coefficient (*ICC(2*)) was 0.73 (*p* < .01).

Participant groups’ *general attitudes* were measured using the items based on General Evaluation Scale (see 50). Participants answered the question, “How does your group evaluate government officials/teachers/doctors in general?” with the following six bipolar adjective pairs (alpha = 0.83): warm-cold, friendly-hostile, suspicious-trusting, positive-negative, admiration-disgust, respect-contempt. Answers were given on a four-point scale without a middle point, and group attitude was generated by averaging the four members’ reported attitudes. For government officials, the intraclass correlation coefficient (*ICC(2*)) was 0.74 (*p* < .01).

Similar to the minimal group paradigm [48,51,52], data on *supportive behaviors* were obtained by asking the participant groups to allocate hypothetical resources to government officials. More allocated resources indicated greater supportive behaviors toward government officials.

Specifically, after attitudes were measured, participant groups were presented with the following instructions: “A well-known nongovernmental organization from a commonwealth in a certain city is planning to use public appraisals to choose 10 paragons from excellent government officials, teachers, and doctors in the city. This organization entrusted us with surveying all kinds of members of the community to fill the allocated quota among the three groups. Now please discuss the allocation of these 10 positions within 15 minutes and write down the group’s consensus on the ballot given to you. Finally, please elect one delegate from your group to put the ballot into the locked ballot box.” This portion of the procedure required the participant groups to allocate a quota of 10 paragons among government officials, teachers, and doctors. Participant groups’ supportive behaviors toward government officials were measured by the number of positions allocated to government officials, which ranged from 0 to 10.

Based on Dovidio et al.’s assessment of *attitude base* [23], we categorized the participant groups into those with affectively-based attitudes and those with cognitively-based attitudes according to evaluative-affective consistency and evaluative-cognitive consistency. Evaluative-affective consistency was measured by calculating the absolute value of the discrepancy between each group’s rankings in general attitude scores and affective component scores, whereas evaluative-cognitive consistency was determined by the absolute value of the discrepancy between the group’s rankings in general attitude scores and cognitive component scores [18,31]. Lower evaluative-affective consistency and evaluative-cognitive consistency scores signified higher evaluative-affective and evaluative-cognitive consistency of group attitudes.

Standardized evaluative-affective consistency and evaluative-cognitive consistency scores were then compared to code two levels of attitude base [23]. Specifically, when the standardized evaluative-cognitive consistency score was greater than the standardized evaluative-affective consistency score, attitude base was coded as affectively-based; otherwise, attitude base was coded as cognitively-based.

The procedure for determining each participant group’s *affective-cognitive consistency* of the attitudes toward government officials followed that set forth by Chaiken and Baldwin [26], Chaiken and Yates [27], Norman [28], Schleicher et al. [29], and Zhou et al. [31]. Specifically, participant groups were rank-ordered separately in terms of affective component scores and cognitive component scores. Affective-cognitive consistency scores were then measured by the absolute value of the discrepancy between the group’s positions in the two rankings. Lower affective-cognitive consistency scores signified higher affective-cognitive consistency of group attitudes.

*Attitude-behavior consistency*, which reflects the relationship between attitudes and behaviors, was quantified by the absolute value of the discrepancy between each group’s standard scores on the scales of general attitudes and behaviors toward government officials [53]. Lower attitude-behavior consistency scores indicated a stronger relation between attitudes and behaviors.

### Results

Analyses testing whether there was an effect of the six different orders of presentation of materials revealed that the orders with which responses to teachers, doctors, and government officials were assessed did not influence the affective components of attitudes, *F*(5, 34) = 1.06, *p* = .40, cognitive components of attitudes, *F*(5, 34) = 1.26, *p* = .30, general attitudes, *F*(5, 34) = 0.39, *p* = .85, and supportive behaviors, *F*(5, 34) = 1.34, *p* = .27, toward government officials.

The primary objective of the current study was to examine the interaction between attitude base and affective-cognitive consistency on the attitude-behavior relation for intergroup consummatory behaviors. Thus, a multiple regression model with attitude-behavior consistency as a dependent variable was used, in which attitude base and affective-cognitive consistency entered by the first step and the interaction between them was added by the second step.

**Figure 1:**
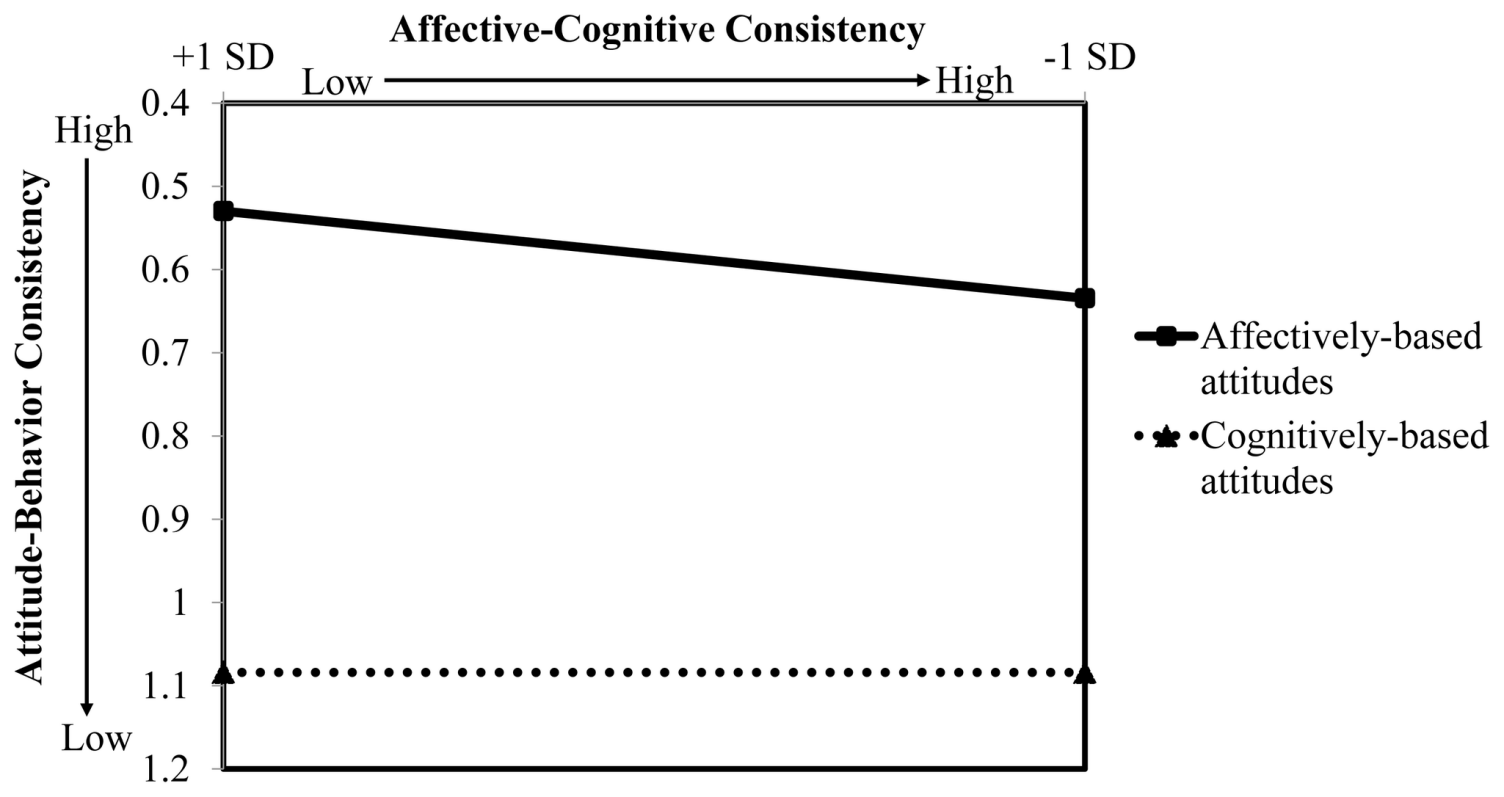
Effects of attitude base and affective-cognitive consistency on intergroup consummatory behaviors in Study 1. This figure was plotted by using the unstandardized regression weights with affective-cognitive consistency along the abscissa at + 1 *SD* from the mean. Lower affective-cognitive consistency scores signified higher affective-cognitive consistency of group attitudes. Similarly, lower attitude-behavior consistency scores indicated a stronger relation between attitudes and behaviors. Results show that affectively-based attitudes predicted intergroup consummatory behaviors more strongly than cognitively-based attitudes regardless of affective-cognitive consistency.

As Table 1 indicates, only attitude base had an effect on attitude-behavior consistency. Attitude-behavior consistency was greater in the affectively-based condition (mean attitude-behavior consistency score = 0.58, *SD* = 0.45) than in the cognitively-based condition (mean attitude-behavior consistency score = 1.08, *SD* = 0.83), *t* (19.02) = 2.15, *p* = .04. To understand the form of this effect, we plotted Figure 1 using the unstandardized regression weights with affective-cognitive consistency along the abscissa at + 1 *SD* from the mean. As illustrated in Figure 1, affectively-based attitudes predicted intergroup consummatory behaviors more strongly than cognitively-based attitudes regardless of affective-cognitive consistency. The main hypothesis in this study was supported.

**Table 1:** Effects of attitude base and affective-cognitive consistency on attitude-behavior relation for intergroup consummatory behaviors in Study 1.

Step	Variables	*B*	*SE B*	*Beta*	*R^2^*	*ΔR^2^*
1	Attitude base	-0.53	0.22	-0.39*	0.142^+^	
	ACC	-0.01	0.02	-0.06		
2	Attitude base	-0.52	0.23	-0.39*	0.144	0.002
	ACC	<0.001	0.02	-0.004		
	Attitude base × ACC	-0.01	0.03	-0.06		

Note. The dependent variable in this regression is attitude-behavior consistency.

ACC = affective-cognitive consistency.

* *p* < .05, ^+^ *p*<.10.

In addition, the present study examined why affective-cognitive consistency did not moderate the role of attitude base in attitude-behavior consistency for intergroup consummatory behaviors by testing the effects of affective and cognitive components on attitudes and behaviors separately. The findings from regression analyses indicated that for groups with affectively-based attitudes, the affective components exerted a significant influence both on general attitudes (in the condition of high affective-cognitive consistency, β = 0.91, *R* ^2^ = 0.83, *p* < .001; in the condition of low affective-cognitive consistency, β = 0.57, *R* ^2^ = 0.32, *p* = .09) and on consummatory behaviors (in the condition of high affective-cognitive consistency, β = 0.72, *R* ^2^ = 0.51, *p* < .001; in the condition of low affective-cognitive consistency, β = 0.58, *R* ^2^ = 0.33, *p* = .08). For groups with cognitively-based attitudes, the cognitive components did exert a significant influence on general attitudes (in the condition of high affective-cognitive consistency, β = 0.96, *R* ^2^ = 0.92, *p* = .04; in the condition of low affective-cognitive consistency, β = 0.59, *R* ^2^ = 0.35, *p* = .05) but not on consummatory behaviors (in the condition of high affective-cognitive consistency, β = 0.47, *R* ^2^ = 0.22, *p* = .53; in the condition of low affective-cognitive consistency, β = -0.23, *R* ^2^ = 0.05, *p* = .49).

### Discussion

As we hypothesized, the results of Study 1 revealed that affectively-based attitudes predicted intergroup consummatory behaviors better than cognitively-based attitudes regardless of affective-cognitive consistency. These findings support previous results showing that affectively-based attitudes often drive consummatory behaviors in the intergroup context [4,23,24,35,46,54], even when affective-cognitive consistency and its interaction with attitude base are taken into consideration.

However, these results appear to contradict Millar and Tesser’s findings that suggested affectively-based attitudes only drive consummatory behaviors in the condition of low affective-cognitive consistency [30]. There are two potential interpretations for these differences. First, Millar and Tesser’s study focused on individuals’ attitudes and behaviors toward puzzles [30], whereas the present study assessed groups’ attitudes and consummatory behaviors toward another group (i.e., undergraduates’ attitudes and supportive behaviors toward government officials). Second, Millar and Tesser [30] manipulated the affective or cognitive basis of the attitudes by asking participants to concentrate on different components (i.e., reasons analysis [55]). In the present research, we studied the relationships among the main basis of attitude (affective or cognitive), affective-cognitive consistency, and attitude-behavior consistency for established intergroup attitudes. Affect may play a much more important role in responses to social groups [4] and in responses by groups [33] than in responses to nonsocial objects (e.g., puzzles) by individuals. In addition, whereas newly developed attitudes toward novel objects (e.g., puzzles) might be still susceptible to different affective and cognitive influences as they form (e.g., different levels of affective-cognitive consistency), existing intergroup attitudes, which are more crystallized, may be more firmly and directly related to the affective base of the attitudes.

Overall, the findings contribute to an understanding of the effects of attitude base and affective-cognitive consistency on the attitude-behavior relation in three important ways. First, the present study extends the earlier work examining the moderating role of affective-cognitive consistency in the effect of attitude base on attitude-behavior consistency to the intergroup context. Within the context of intergroup attitudes, consummatory behaviors are primarily related to affectively-based attitudes, regardless of affective-cognitive consistency.

Second, this study sheds light on why affective-cognitive consistency does not influence the effects of affectively-based and cognitively-based attitudes on intergroup consummatory behaviors. No matter what the level of affective-cognitive consistency is, the affective components exert a significant influence both on general attitudes and on consummatory behaviors in the affectively-based condition. By contrast, the cognitive components exert a significant influence on general attitudes but not on consummatory behaviors in the cognitively-based condition. As a result, attitude-behavior consistency varies between affectively-based and cognitively-base attitudes in both conditions of affective-cognitive consistency.

Third, extending previous work with individuals as participants, the current study collected group data to explore the effects of attitude base and affective-cognitive consistency on the attitude-behavior relation for consummatory behaviors. In addition, this study provides measurements of group attitudes and behaviors. By utilizing these measures, future intergroup research can use group data to validate the conclusions obtained from individual assessments.

Nevertheless, the present study only focused on intergroup consummatory behaviors. Further investigation is necessary for the moderating role of affective-cognitive consistency in the effects of affectively-based and cognitively-based attitudes on intergroup instrumental behaviors.

## Study 2

Study 2 examined the effects of attitude base and affective-cognitive consistency on the attitude-behavior relation for intergroup instrumental behaviors. In Study 1, participants’ expressed support for government officials was a preference representing consummatory behaviors that had no direct consequences for participants. Instrumental behaviors, by contrast, are performed to achieve a specific objective. Because it is unlikely that participants would believe that their responses would have a direct and immediate effect on government officials, we altered the paradigm in Study 2 to examine responses to a group that was present and which participants might be able to influence in the experimental setting.

Following Saguy, Dovidio, and Pratto’s protocol [56], all participant groups were led to believe that they were members of a supervised group by informing them that the other (supervisory) group would decide how to allocate resources and extra rewards during a team task. Moreover, we offered the participant groups an opportunity to interact with the supervisory group so that they could form attitudes and demonstrate supportive behaviors.

Supportive behaviors were assessed by asking the participant groups to allocate limited rewards to the supervisory group. And the supervisory group would be made aware of the allocation. These behaviors were taken as instrumental behaviors because the allocation given to the supervisory group could adversely influence the participant groups’ own rewards so that the participant groups performed these supportive behaviors for achieving their goals (i.e., pursuing more interests in the experiment). It is important to note that this measure of behaviors did not affect the participant groups’ status as supervised groups since they were told that their allocation would only be used as a reference to determine the supervisory group’s final reward.

According to much previous work, we hypothesized that cognitively-based attitudes would be stronger predictors of intergroup instrumental behaviors than affectively-based attitudes (see 7,20,24). However, to the extent that intergroup orientations have a generally strong affective component [4,34], affective-cognitive consistency may also play a role in this case. In particular, the present study might expect the moderating effect of affective-cognitive consistency, which is more analogous to Millar and Tesser’s research [30]. That is, cognitively-based attitudes may relate more strongly to intergroup instrumental behaviors when the cognitive and affective components of the attitudes are high in consistency than when they are low in consistency. When affective-cognitive consistency is low, the predominant role of affect in intergroup orientations would weaken the effects of cognitively-based attitudes. In contrast, affectively-based attitudes would have only a weak correlation with intergroup instrumental behaviors, regardless of affective-cognitive consistency.

Moreover, similar to Study 1, Study 2 also explored the effects of the affective and cognitive bases and affective-cognitive consistency of an intergroup attitude on the two elements of attitude-behavior consistency – the general attitude and the instrumental behavior – to understand the mechanisms involved. We expected that for cognitively-based attitudes, the cognitive components may predict general attitudes toward the other group regardless of affective-cognitive consistency because they represent the dominant component of the attitudes, but inconsistency between cognitive and affective components of the attitudes may undermine the relationship of the cognitive components to instrumental behaviors. However, because affectively-based attitudes are hypothesized primarily to predict consummatory rather than instrumental behaviors, we expected that for affectively-based attitudes, the affective components may relate to general attitudes but be less strongly associated with instrumental behaviors, no matter what the level of affective-cognitive consistency is.

### Method

#### Ethics Statement

All participants gave a written informed consent before this experiment, and their responses in the current study are all anonymous. In addition, this study was approved by the Institutional Review Board (IRB) of Institute of Psychology, Chinese Academy of Sciences.

#### Participants

Participants were 152 undergraduate volunteers (66 men and 86 women) at a Chinese university ranging in age from 18 to 33 years (mean = 21.75 years). They were randomly assigned to one of 38 groups, each of which consisted of four members.

#### Procedure

Participant groups were given a short oral introduction describing the purpose of the experiment as “a team cooperation study.” They were required to reach unanimous group opinions in a series of discussions without the use of a ballot. Each large team consisting of a supervisory group and two task groups were charged with performing a community programming task, in which they must discuss and choose ten essential facilities for a new community from a total of twenty options (e.g. greenbelt, shop, food market and drugstore). This community programming task would be divided into two stages. At the first stage, the task group should list the selected ten facilities on an answer sheet after fifteen-minute discussion. Then at the second stage, the task group would be permitted to check the answers within five minutes by using a help manual that includes the programming experts’ analyses of all options and can greatly improve the task performance. Each participant group acting as a task group (i.e., a supervised group) was told that the reward amount they were given would depend on their performance which would be decided by the numbers of their choice consistent with the programming experts’ answers. And then an additional bonus would be allocated by the supervisory group if the performance of both task groups was acceptable. Furthermore, the supervisory group would decide how to distribute the help manual between two task groups.

After participant groups finished the first stage of the community programming task, they were informed that they could not use the help manual in the following feedback stage because the supervisory group had decided to allocate it to another task group. Each participant group asked to appeal this decision, but all requests were refused by the supervisory group. After the programming tasks were completed, participant groups were asked to spend 15 minutes discussing their group’s status, the feelings they experienced when they thought about the supervisory group, and the typical characteristics exhibited by the supervisory group. Then participant groups’ attitudes toward the supervisory group and the affective and cognitive components of these attitudes were measured. Afterwards, participant groups’ supportive behaviors were assessed.

In fact, the supervisory group and another task group were both fictitious in this experiment. All interactions between the participant groups and the supervisory group took place indirectly through a female experimenter. Similarly, the reward amount depending on the performance was bogus. Each participant got 20 yuan regardless of group performance.

At the end of the experiment, each participant completed a questionnaire that included an item about the purpose of the experiment. None of the participants was able to identify its true purpose. Moreover, all participant groups believed that they had been supervised in low status so that the manipulation of group status was successful.

#### Measures

The *affective components of group attitudes* toward the supervisory group were assessed using three items that asked the members of the participant groups to answer the question, “How does your group feel about the supervisory group?” with the following bipolar adjective pairs (alpha = .89): happy-unhappy, relaxed-angry, and loving-hateful. Answers were given on a four-point scale without a middle point. Group attitude was calculated by averaging the four members’ reports.

The *cognitive components of group attitudes* were assessed by asking, “What does your group think of the supervisory group?” Participants answered this question using a four-point scale and the following three bipolar adjective pairs (alpha = .91): responsible-irresponsible, competent-inept, and helpful-useless. The four group members’ scores were then averaged to obtain the index of group attitude.

Participant groups’ *general attitudes* were measured by asking, “How does your group evaluate the supervisory group in general?” with the following six bipolar adjective pairs (alpha = 0.91): warm-cold, friendly-hostile, suspicious-trusting, positive-negative, admiration-disgust, and respect-contempt. Once again, answers were given on a four-point scale without a middle point. Group attitude was calculated by averaging the four members’ reported attitudes.

Although the low intraclass correlation coefficients for the above three measures (*ICC(2)s* < .60) indicated large degrees of intra-group variances among group members, Barsade [40] suggested the reports of groups’ subjective perceptions (e.g., attitudes and emotion) required much lower consistency within the group than other reports of groups’ relatively objective characteristics (e.g., group atmosphere and cooperative efficiency). Therefore, in this study, the average of the four group members’ attitudinal reports was still used as the indexes of group affective components, cognitive components and general attitudes, because even weak interdependencies can adversely affect statistical inferences based on assumptions of independence of observations.

As in Study 1, data on *supportive behaviors* were obtained by asking the participant groups to allocate hypothetical rewards to the supervisory group. More allocated rewards indicated greater supportive behaviors toward the supervisory group.

Specifically, after attitudes were measured, participant groups were presented with the following instructions: “The supervisory group’s final bonus will be a weighted average of the rewards given by your group, another task group and the experimenter interacting with them. In the next five minutes, please discuss how much of a reward your group thinks the supervisory group should get. The amount of reward must be between 0 and 60 yuan, and *it will possibly influence your group*’s *own interests* because after the supervisory group knows the reward your group gives to them, they will allocate an additional bonus between your group and another task group since the performance of both task groups is acceptable.” Participant groups’ supportive behaviors were then measured by the reward amounts they gave to the supervisory group, ranging from 0 to 60.

The procedures for determining each participant group’s *attitude base, affective-cognitive consistency, and attitude-behavior consistency* were the same as those used in Study 1.

### Results

The objective of this study was to investigate how affective-cognitive consistency impacts the role of attitude base in the attitude-behavior relation for intergroup instrumental behaviors. As in Study 1, a multiple regression model using attitude-behavior consistency as a dependent variable was constructed. In the first step, attitude base and affective-cognitive consistency were included in this model. In the second step, the interaction between attitude base and affective-cognitive consistency was added into the model.

**Figure 2:**
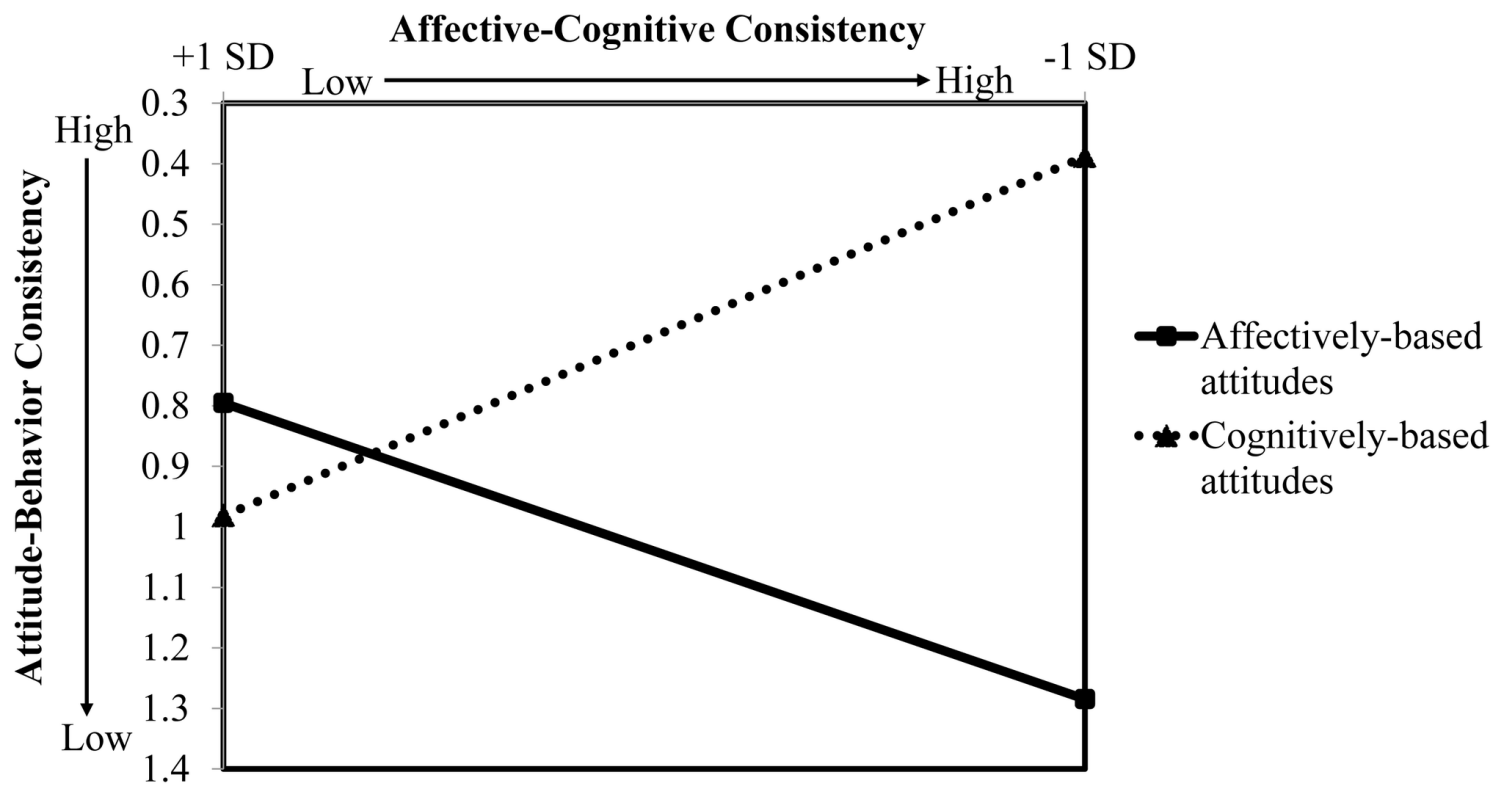
Effects of attitude base and affective-cognitive consistency on intergroup instrumental behaviors in Study 2. This figure was plotted by using the unstandardized regression weights with affective-cognitive consistency along the abscissa at + 1 *SD* from the mean. Lower affective-cognitive consistency scores signified higher affective-cognitive consistency of group attitudes. Similarly, lower attitude-behavior consistency scores indicated a stronger relation between attitudes and behaviors. Results representing the moderating role of affective-cognitive consistency in the effects of affectively-based and cognitively-based attitudes on intergroup instrumental behaviors show that cognitively-based attitudes predicted intergroup instrumental behaviors more strongly than affectively-based attitudes only when affective-cognitive consistency was high.

The results shown in Table 2 indicated that the interaction between attitude base and affective-cognitive consistency had a significant effect on attitude-behavior consistency. Figure 2 representing the form of this interaction revealed that for groups with cognitively-based attitudes, attitude-behavior consistency was greater when affective-cognitive consistency was high (mean attitude-behavior consistency score = 0.26, *SD* = 0.24) than when affective-cognitive consistency was low (mean attitude-behavior consistency score = 0.83, *SD* = 0.71), *t*(16.99) = -2.62, *p* = .02; for groups with affectively-based attitudes, in contrast, attitude-behavior consistency did not vary between the condition of high affective-cognitive consistency (mean attitude-behavior consistency score = 1.13, *SD* = 0.72) and the condition of low affective-cognitive consistency (mean attitude-behavior consistency score = 0.80, *SD* = 0.69), *t*(17) = 0.89, *p* = .39. In other words, cognitively-based attitudes predicted intergroup instrumental behaviors more strongly than affectively-based attitudes only when affective-cognitive consistency was high. The moderating effect hypothesis in this study was supported.

**Table 2:** Effects of attitude base and affective-cognitive consistency on attitude-behavior relation for intergroup instrumental behaviors in Study 2.

Step	Variables	*B*	*SE B*	*Beta*	*R^2^*	*ΔR^2^*
1	Attitude base	0.43	0.24	0.31^+^	0.083	
	ACC	0.02	0.02	0.14		
2	Attitude base	0.32	0.23	0.23	0.210*	0.127*
	ACC	0.04	0.02	0.36^+^		
	Attitude base × ACC	-0.09	0.04	-0.44*		

Note. The dependent variable in this regression is attitude-behavior consistency.

ACC = affective-cognitive consistency.

* *p* < .05, ^+^ *p*<.10.

Moreover, the present study tested the reasons why affective-cognitive consistency moderated the effects of affectively-based and cognitively-based attitudes on intergroup instrumental behaviors. The findings from regression analyses showed that for groups with cognitively-based attitudes, the cognitive components exerted a significant influence both on general attitudes (β = 0.99, *R* ^2^ = 0.98, *p* < .001) and on instrumental behaviors (β = 0.98, *R* ^2^ = 0.96, *p* < .001) in the condition of high affective-cognitive consistency, whereas in the condition of low affective-cognitive consistency, the cognitive components exerted a significant influence on general attitudes (β = 0.94, *R* ^2^ = 0.89, *p* < .001) but not on instrumental behaviors (β = 0.14, *R* ^2^ = 0.02, *p* = .64). However, for groups with affectively-based attitudes, the affective components exerted a significant influence on general attitudes (in the condition of high affective-cognitive consistency, β = 0.93, *R* ^2^ = 0.87, *p* < .001; in the condition of low affective-cognitive consistency, β = 0.85, *R* ^2^ = 0.73, *p* = .07) but not on instrumental behaviors (in the condition of high affective-cognitive consistency, β = -0.08, *R* ^2^ = 0.01, *p* = .78; in the condition of low affective-cognitive consistency, β = 0.76, *R* ^2^ = 0.58, *p* = .11), regardless of affective-cognitive consistency.

### Discussion

Consistent with the premise that intergroup orientations have a strong affective component, Study 1 found that attitude-behavior consistency was greater for consummatory behaviors when the basis of the attitudes was affective than cognitive. In contrast, Study 2, which focused on attitude-behavior consistency for intergroup instrumental behaviors, found that the attitude-behavior relationship was particularly strong for cognitively-based attitudes, but only when affective-cognitive consistency of the attitudes was high. While these results generally support the finding in previous work of the important potential moderating role of affective-cognitive consistency, the nature of our moderation is opposite to what Millar and Tesser obtained [30]. Their study revealed that cognitively-based attitudes had a stronger influence on instrumental behaviors (i.e., play the puzzle games for getting a high score in the following puzzle-solving test) when affective-cognitive consistency was lower.

However, as we posited, the prominent affective nature of intergroup orientations [4,34] can shape the dynamics of intergroup instrumental behaviors in different ways. Millar and Tesser [30] created affectively-based and cognitively-based attitudes toward novel objects (puzzles); by contrast, we examined group attitudes held toward another group which were formed through intergroup interactions. In addition, we measured whether participants’ attitudes toward the supervisory group were predominantly affectively-based or cognitively-based. We hypothesized that affect plays a particularly important role in intergroup orientations, which we demonstrated directly with consummatory behaviors in Study 1. In Study 2 with intergroup instrumental behaviors, affect also played an important role, but more indirectly. Because of the hypothesized primacy of affect in intergroup relations, affective-cognitive inconsistency appeared to undermine the general importance of cognitively-based attitudes for instrumental behaviors. That is, whereas the cognitive components of the attitudes were still significantly related to general attitudes, they did not predict instrumental behaviors in this case – thus weakening attitude-behavior consistency. However, when the affective and cognitive components of the attitudes were consistent, cognitively-based attitudes were significantly related to both general attitudes and intergroup instrumental behaviors, producing the highest level of attitude-behavior consistency in Study 2.

This overall pattern of findings may apply to other contexts in which affect plays a primary role. For instance, when ownership of an object [57] or even an idea [58] is involved, people have a strong affective attachment. This may help account for the findings of Zhou et al.’s research [31], which examined consumers’ purchasing behaviors and paralleled those results obtained in the present study: Cognitively-based attitudes were better predictors of instrumental behaviors than affectively-based attitudes when affective-cognitive consistency was high, but cognitively-based and affectively-based attitudes exerted a roughly equal influence when affective-cognitive consistency was low.

Some may think that it is not very important to show the different effects of affectively-based and cognitively-based attitudes on the following behaviors when the affective and cognitive components of the attitudes are consistent. But in fact, as discussed above, unfolding the distinct impacts of affectively-based and cognitively-based attitudes, even only in high affective-cognitive consistency condition, can theoretically help understand the primary role of affect in the intergroup context. Moreover, in practice for behavior interventions, it is quite essential to identify which component will predict the following behaviors, because the behaviors based on a certain component (e.g., affective component) can be effectively changed by altering this component (e.g., presenting the affective information; [24]). 

Thus, Study 2 complements Study 1 by examining intergroup instrumental behaviors rather than consummatory behaviors and triangulates on the important but different role of affect in predicting distinct types of behaviors and the ultimate difference of attitude-behavior consistency.

## General Discussion

Despite the historical focus in the study of intergroup relations on improving intergroup attitudes [35], the relationship between intergroup attitudes and behaviors is modest [3,4]. Understanding the dynamics of attitude-behavior consistency can thus have both theoretical and practical implications for intergroup relations. The present research attempted to integrate research and theory from the study of intergroup relations and from the literature on attitudes generally – two bodies of work that have developed in relatively independent ways [59].

Building on the intergroup work, theoretically we emphasize the particularly pronounced role that affect plays in intergroup attitudes relative to other types of attitudes. Empirically, we focus on intergroup consummatory (Study 1) and instrumental (Study 2) behaviors in the context of group decision making. From the literature on general attitudes, we consider the importance of distinguishing between affective and cognitive basis of an attitude and affective-cognitive consistency [30]. In both studies, we examined the relationship of these factors to attitude-behavior consistency and to the separate attitudinal and behavioral components [47].

Our findings offer several specific insights into work on the attitude-behavior relationship. First, our research generally supports previous work that considers both attitudes generally [7,20] and intergroup attitudes [24], and identifies the importance of distinguishing the primary basis (affective or cognitive) of an attitude in relation to the nature of the behavior (consummatory or instrumental) in question. Our findings are consistent with previous work showing that affectively-based attitudes often drive consummatory behaviors [4,23,24,35]. Second, our research extends this line of work in intergroup attitudes by further investigating the moderating role of affective-cognitive consistency in how affectively-based and cognitively-based attitudes relate to attitude-behavior consistency for different types of intergroup behaviors.

Third, taken together, our two studies point to the primacy of affective reaction in intergroup orientations. In Study 1, affectively-based attitudes were associated with greater attitude-behavior consistency than cognitively-based attitudes for intergroup consummatory behaviors, regardless of affective-cognitive consistency. In Study 2, cognitively-based attitudes showed high attitude-behavior consistency for intergroup instrumental behaviors, but only when affective-cognitive consistency was high. And fourth, the present research clarifies how these factors combine to influence attitude-behavior consistency. In general, we found that the attitude-behavior relationship is eroded primarily because of the weaker relationship of affective or cognitive components to behaviors than to general attitudes.

In addition, the current work provides measurements of *group* attitudes and behaviors toward outgroup, and develops a new paradigm through which group attitudes and behaviors can be formed following some intergroup interactions. In an influential article entitled “Whatever Happened to the Group in Social Psychology?”, Steiner [60] observed that “by the 1960’s social psychology had become much more individualistic. Interest in the group as a system had waned and research was generally focused on intraindividual events or processes that mediate responses to social situations” (p. 94). Steiner warned that such a strict individualistic focus could not capture the transcendent influence of group processes on social life. Indeed, work in the interindividual-intergroup discontinuity effect [33] demonstrates the fundamental differences between groups and individuals. Intergroup reactions have a stronger affective component (e.g., fear) and are more competitive and exploitative. Thus, our group-based paradigm may be particularly useful for future research studying group-based attitudes, behaviors, and relations.

We note, however, that inferences about the causal direction of the relationships we observed in our studies are limited by the fact that we measured rather than manipulated whether attitudes were affectively-based or cognitively-based. As previous research demonstrates, it is possible to experimentally create novel attitudes toward stimuli that vary in the strength of their affective or cognitive components [30,31] or to vary the salience of these components of existing attitudes [24]. Our results may thus apply primarily to relatively strongly-held attitudes toward groups. Nevertheless, in Study 2, the laboratory-based groups were used without a previous intergroup history.

Future research might thus productively extend the current work in experimental directions. One possibility might be to create minimal group relations [61,62] to study the roles of affect and cognition in intergroup attitudes, behaviors, and attitude-behavior consistency. Under minimal group conditions, group memberships are determined on relatively meaningless bases (e.g., assignment ostensibly based on whether people underestimate or overestimate dots on a page), without direct functional interdependence. Such assignment produces spontaneous differences in affective reactions [63], as well as in cognitive responses (accentuating with-group similarities and between-group differences [62]). It is possible that there may be greater balance in the influences of affectively-based and cognitively-based attitudes with minimal groups, in which the absence of functional relationships is less likely to produce strong and differentiated emotional reactions [64]. In addition, it may be easier to manipulate the affective or cognitive basis of the attitudes toward a minimally-created group than to manipulate the affective or cognitive basis of the attitudes toward existing groups, which may be well-crystallized because of the ongoing nature of intergroup relations. A minimal-group paradigm might also allow direct tests between attitudes and behaviors assessed individually or collectively by participants in groups.

Another direction for future research is to directly examine the role of affective-cognitive consistency for affective and cognitive interventions to improve intergroup behaviors. Understanding the influence of affective-cognitive consistency can help shape more effective interventions [24,65]. Thus, the current research suggests several potential directions for understanding and changing intergroup attitudes, behaviors, and attitude-behavior consistency.

In conclusion, this research provides insight into the moderating role of affective-cognitive consistency in the effects of affectively-based and cognitively-based attitudes on intergroup consummatory and instrumental behaviors and supports an important mechanism by which this moderating role of affective-cognitive consistency occurs. Future research can do much to enrich our findings and aid in our understanding of the impacts of affective and cognitive interventions for increasing the positive intergroup behaviors.
